# ESHRE position paper: international limits on the number of offspring per gamete donor

**DOI:** 10.1093/humrep/deag108

**Published:** 2026-07-08

**Authors:** Lucy Frith, Johanna Tassot, Aisling Ahlstrom, Dinka Pavičić Baldani, Carlos Calhaz-Jorge, Gueorgui Nikolov, Petra Thorn, Nathalie Vermeulen, Jackson Kirkman-Brown

**Affiliations:** Centre for Social Ethics & Policy, Department of Law, School of Social Sciences, University of Manchester, Manchester, UK; European Society of Human Reproduction and Embryology, Central Office, Strombeek-Bever, Belgium; IVIRMA Global Research Alliance, Livio Gothenburg, Gothenburg, Sweden; University Clinical Hospital Zagreb, School of Medicine, University of Zagreb, Zagreb, Croatia; Obstetrics and Gynaecology Department, Faculdade de Medicina, Universidade de Lisboa, Lisbon, Portugal; ReproBioMed Medical Centre, Sofia, Bulgaria; Private Practice, Moerfelden, Germany; European Society of Human Reproduction and Embryology, Central Office, Strombeek-Bever, Belgium; Centre for Human Reproductive Science, School of Medical Sciences, College of Medicine & Health, University of Birmingham, Birmingham, UK; Birmingham Women’s Fertility Centre, Birmingham Women’s Hospital, Edgbaston, Birmingham, UK

**Keywords:** gamete donation, donor conception, donor offspring limits, reproductive ethics, regulation

## Abstract

**STUDY QUESTION:**

How should the number of offspring per gamete donor be regulated at an international level?

**SUMMARY ANSWER:**

The European Society of Human Reproduction and Embryology (ESHRE) recommends introducing a European Union (EU)-wide limit on the number of families per gamete donor, starting at 50 families and gradually reducing it to a maximum of 15 families or lower, supported by an EU-wide donor registry to monitor compliance.

**WHAT IS KNOWN ALREADY:**

Most European countries impose national limits on the number of offspring or families per donor, but these limits vary widely and are not always enforced. Cross-border movement of patients and the export of donor gametes between countries mean that large donor sibling groups can still emerge despite these restrictions. What matters to donor-conceived people is the total number of donor siblings, regardless of which country they are in, making a transnational limit the only relevant mechanism that can address this issue.

**STUDY DESIGN, SIZE, DURATION:**

This position paper sets out principles relevant to international donor offspring limits and the position and policy recommendations of ESHRE, focusing on the EU level.

**PARTICIPANTS/MATERIALS, SETTING, METHODS:**

This ESHRE position paper was developed by a multidisciplinary expert working group. Recommendations are supported by data from the literature, where available. The first version was published for stakeholder review in November 2025, with 45 completed review forms received from organizations representing donor-conceived people, families built through donor conception and infertility patients, national fertility societies, researchers, professionals from different disciplines, and gamete banks. The paper was then revised on the basis of the stakeholder review.

**MAIN RESULTS AND THE ROLE OF CHANCE:**

ESHRE considers that the wellbeing of donor-conceived people should have the highest priority when determining limits, while also balancing the wellbeing of prospective parents. Limits should be set for the number of families rather than individual children. ESHRE proposes a phased introduction starting at 50 families per donor, being reduced to a maximum of 15 families or lower, alongside a 20-year cap on distributing gametes to new families after the first donation. National limits should still be upheld where they are lower, and donors should be able to set their own lower personal limit. Compliance should be monitored through an EU-wide donor registry or, failing that, national registries, with gamete banks (including non-EU banks exporting into the EU) obliged to enforce the limit, donors required to declare all previous donations, and families counted as potential live births unless confirmed otherwise.

**LIMITATIONS, REASONS FOR CAUTION:**

The evidence base on the psychosocial impact of large donor sibling groups is small and developing, and definitive evidence of harm is limited. Research on the preferences of donor-conceived people, donors, and recipients regarding offspring limits is scarce and inconclusive. These recommendations therefore take a precautionary approach, and the proposed 15-family limit should be reviewed in light of developing knowledge during the transition period.

**WIDER IMPLICATIONS OF THE FINDINGS:**

An EU-wide limit would represent a feasible first step towards an international limit. An EU-wide donor registry could potentially serve secondary purposes such as supporting tracing in case of the diagnosis of a serious genetic condition and giving access to information to donors and donor-conceived people.

**FUNDING:**

Support for the working group was provided by ESHRE.

**DISCLOSURES:**

L.F. and C.C.-J. report travel support from ESHRE. D.P.B. reports speakers' fees from Merck, Ferring, Gedeon Richter and MSD, travel support from ESHRE, and a position as the president of the Croatian Society for Gynaecological Endocrinology and Human Reproduction. A.A. reports speakers’ fees from Merck Healthcare KGaA and Ferring, travel support from ESHRE, passive shareholder interest in Inception Midco 1. S.à r.l., membership in a subgroup of the European Medical Devices Coordination Group, and a position as vice chair of the EXPAMED panel on obstetrics, gynaecology and reproductive medicine. G.N. reports speakers’ fees from Gedeon-Richter and Organon, travel support from ESHRE, and a position as past chairman and member of the Executive Committee of the Bulgarian Association for Human Reproductive Embryology. P.T. reports speakers’ fees and travel support from Ferring and Gedeon Richter and a position as a board member on the Arbeitskreis donogene Insemination. J.K.-B. reports speakers’ fees from Ferring, IBSA, Merck and CooperSurgical, travel support from Merck and ESHRE, an associate editor position for the journal Andrology, and a position as chair of the scientific advisory committee of the Association for Reproductive and Clinical Scientists (ARCS). The remaining authors (J.T. and N.V.) have nothing to declare.

**TRIAL REGISTRATION NUMBER:**

n/a.

## Introduction

A significant number of people make use of donated gametes (sperm or oocytes) to conceive (The European IVF Monitoring Consortium for the European Society of Human Reproduction Embryology *et al.*, 2025). Donor gametes may be used for a variety of reasons, such as impaired or absent ovarian function or sperm production, age-related fertility decline, relationship status (i.e. same-sex couples or single women), among others. While most European countries impose national limits on the number of offspring or families that can be created using gametes from a single donor in their country (see Supplementary Data File S1), these limits are not always enforced, and there is no agreed international limit. In Europe, the movement of patients and the export of donor gametes to other countries are common practices. For example, a study from 2014 found that 63% of inseminations with donor sperm in Belgium were carried out with donor sperm imported from Denmark (Thijssen *et al.*, 2014). In this context, the lack of an international limit on gamete use can lead to large groups of donor-conceived people from the same donor spread throughout multiple countries.

Recently, the national ethics councils of four Nordic countries (Denmark, Finland, Norway, and Sweden) published a joint statement calling for an international limit on the number of children per sperm or oocyte donor (Swedish National Council on Medical Ethics *et al.*, 2025). The topic has also received wide public attention following a court case in 2023 in which a Dutch sperm donor was prohibited from making further donations after it was discovered that at least 550 people had been conceived using his sperm via donations to several clinics, sperm banks, and private donations (‘Dutch court orders sperm donor to stop after 550 children’, 2023), and a more recent case whereby sperm from a donor with a rare cancer-causing gene was used for the conception of at least 197 children (Conrad and Schülke, 2025).

This position paper describes the relevant principles that should be considered in debates surrounding international limits on the number of offspring born from a single gamete donor and sets out the position of the European Society of Human Reproduction and Embryology (ESHRE) on this topic. It proposes a number of policy recommendations on how to regulate the number of offspring per gamete donor, focusing on the European Union (EU) level.

This position paper focuses specifically on gamete donation. Embryo donation is excluded from the scope of this position paper, except in the case that it involves embryos created with the use of donor gametes. The recommendations focus on setting international limits and on creating monitoring and enforcement mechanisms to ensure compliance with set limits. While not within the scope of this position paper, it is also important that all parties receive comprehensive information on the limits that apply, if any, where they are donating or receiving treatment and the reasoning behind them and that they are counselled about the implications and the possibility that applicable limits may be exceeded in the absence of a worldwide limit. More detailed recommendations on information and counselling can be found in the ESHRE Good Practice Recommendations on information provision for those involved in reproductive donation (ESHRE Working Group on Reproductive Donation *et al.*, 2022).

There are implications of the language used when discussing donor conception since different terms raise different connotations on the relations between the parties involved. Some organizations representing donor-conceived people do not agree with the use of the terms ‘donor’ and ‘donation’, preferring, for instance, terms like ‘genetic father’ or ‘biological father’ rather than ‘sperm donor’ (Spenderkinder, 2021). In contrast, donors and recipients may prefer terms that are not associated with family relations. Recognizing that there is no terminology preferred by all parties, we have opted to use the terms ‘donor’, ‘donor offspring’, ‘donor-conceived people’, and ‘donor siblings’ throughout this position paper, since these terms are widely used and will be understood by most readers. The term ‘donor siblings’ refers to donor-conceived people from the same donor. We also acknowledge that terms like ‘supply’ and ‘shortage’ may not be deemed appropriate by all in relation to gametes. However, we decided to use these terms when discussing the implications of donor offspring limits to be aligned with the terminology used in the EU Substances of Human Origin (SoHO) Regulation.

## Aspects to take into account when determining donor offspring limits

### Psychosocial aspects

There is a small but growing evidence base on the psychosocial aspects of gamete donation, with some studies indicating challenges of large donor sibling groups for donor-conceived people and donors. While the evidence for psychosocial harm is limited, there is also no reassuring evidence that large donor sibling groups are not problematic. Therefore, potential risks that have not yet been widely studied are taken into account in this position paper.

#### Contact between donor siblings

Many donor-conceived people find connections with donor siblings important, leading them to seek contact with each other through direct-to-consumer genetic (DNA) testing (i.e. genetic testing that can be accessed directly online, without the involvement of medical professionals) and donor sibling registries with the aim to find out the number of donor siblings and/or establish meaningful relationships (Indekeu *et al.*, 2021). This contact is often perceived as positive and rewarding (Jadva *et al.*, 2010; Persaud *et al.*, 2017; Frith *et al.*, 2018; Scheib *et al.*, 2020; Hertz, 2022; Indekeu *et al.*, 2022). However, if the search for donor siblings leads to the discovery of a large donor sibling network, it can be overwhelming, distressing, and difficult to navigate (Indekeu *et al.*, 2022; Bolt *et al.*, 2023; Donor Conceived UK, 2025). Moreover, not all donor-conceived people are interested in a relationship with donor siblings. For these people, being found and contacted by a high number of donor siblings may be difficult. It is also important to take into account the time span and geographical spread of donations. Donated gametes can be cryopreserved and used over long periods of time, depending on storage time limits, potentially leading to large age gaps between donor siblings. Furthermore, the export of gametes can lead to a spread of donor siblings across the world. While some donor-conceived people may prefer a geographical spread rather than a concentration of donor siblings in the same area, far distances and language barriers can make it more difficult to build a meaningful relationship for those who seek contact (Bolt *et al.*, 2023).

#### Contact between donor and donor offspring

While contact with the donor was not an option for most donor-conceived people a few decades ago, many countries now only allow identity-release donation, giving donor-conceived people access to identifying information about the donor at a certain age (Calhaz-Jorge *et al.*, 2024). Even if this possibility is not provided, donor-conceived people are often able to identify the donor through online genetic testing and internet research (Crawshaw *et al.*, 2015; Gilman *et al.*, 2024). For those who hope to establish contact with the donor, a large number of donor offspring potentially lowers the chances of establishing such contact. For a donor, it might be difficult to respond to each contact request in a thoughtful way if there are a great number of offspring, let alone invest in an ongoing relationship with each of them if desired. Just like with donor-conceived people, the discovery of a large number of donor offspring might feel overwhelming to donors. Donors might also be concerned about the impact of large offspring numbers on their own children. There are currently no studies indicating whether donors are more or less comfortable about their donation being used abroad or in their own country. While a certain distance between the donor and the recipient family may be perceived as beneficial to preserve the family unit, a geographical spread across countries could create difficulties for donors and offspring who are interested in contact with each other. Moreover, if donated gametes are cryopreserved and only used after a long time span, there is an increased risk that the donor is already deceased at the time when the offspring is old enough to consider seeking contact.

#### Uncertainty

The lack of an international limit on the number of offspring that can be conceived from a donor creates uncertainty for all parties. Donors cannot foresee the implications of their donation at the time when they donate and have to accept the possibility that they could always be contacted by additional donor offspring. Likewise, for donor-conceived people and parents, the gradual discovery of more donor siblings over time and the uncertainty regarding the total number can be difficult to handle and cause stress (Indekeu *et al.*, 2022; Devlin, 2024). Moreover, many donor-conceived people wonder if they are genetically related to other people in their immediate environment and are concerned over the risk of consanguinity with donor siblings (Ravelingien *et al.*, 2013). These concerns can be expected to be stronger in a context where large numbers of donor siblings are likely due to an absence of effective limits.

#### Sense of commodification

Some donor-conceived people have expressed feeling ‘mass-produced’ or like a commodity when finding out about large numbers of donor siblings (Devlin, 2024; Kramer, 2024).

### Access to treatments with donor gametes

If international donor limits similar to current national limits are implemented, fewer families can be created per donor, and unless donor recruitment can be increased quickly and substantially, patients will likely have to wait longer until they can get access to donor gametes, or they may not be able to access donor gametes at all. Even if the reduction in the numbers of families per donor can be compensated for by recruiting more donors, the costs of fertility treatments with donor gametes are expected to increase, since the costs of recruiting and screening a gamete donor are distributed over fewer recipients. Longer waiting times and higher costs will increase inequalities in access to fertility care and leave some patients unable to fulfil their wish for a child, especially in the absence of public funding for treatments with donor gametes. An increase in the number of people who will resort to private donations outside of gamete banks and fertility clinics is also to be expected, which can be associated with risks for all parties, such as the transmission of infections or serious genetic conditions, lack of clarity on the legal parental status of the donor and intended parents, and challenges in upholding the right of donor-conceived people to access information on their genetic origin where this is provided for in the national legislation (Human Fertilisation and Embryology Authority).

### Genetic aspects

#### Consanguinity

The introduction of donor offspring limits at national levels has historically been justified by concerns over consanguinity, i.e. concerns that donor siblings might form romantic and/or sexual relationships without knowing about their genetic relation. Several studies have modelled the risk of consanguinity between donor-conceived people and identified very low risk levels, especially in the context of the international use of gametes where there is a wide geographical spread of donor siblings (Janssens *et al.*, 2015). The risk of consanguinity is likely to be reduced even further by the increasing openness of parents to inform their children that they are donor-conceived and of donors to inform their own children about their donations, as well as increased access to genetic testing. Therefore, consanguinity is no longer the main concern.

#### Propagation of genetic diseases

Cases of donors passing on a serious genetic condition to many offspring receive wide media attention and often fuel debates on donor offspring limits. However, for an individual recipient, the risk that a donor has a serious genetic condition is not related to such limits. From a public health perspective, there is also no relation between the number of offspring per donor and the overall prevalence of genetic conditions in the population (Janssens *et al.*, 2015). While genetic conditions can never be fully prevented, the risk of genetic conditions is, in fact, often lower for donor-conceived people than for other people, as donors undergo a family history review of genetic conditions and many (but not all) donors undergo genetic screening. In the EU, the SoHO Regulation will require clinics or banks as of August 2027 to routinely test donors for potentially life-threatening, disabling, or incapacitating autosomal recessive genetic conditions with a significant prevalence in the donor population to the extent that national legislation allows for such testing. From the perspective of preventing genetic disease transmission, spreading the use of a donor over a certain time period may be beneficial because genetic conditions in the donor might be detected after the first donation, allowing for the permanent deferral of donors before the offspring limit is reached, hence reducing the number of children potentially affected.

### Preferences of affected groups

Research on the preferences of donor-conceived people, donors, and recipients regarding donor offspring limits is scarce and inconclusive. In a Swedish study surveying gamete donors in 2013, 5–8 years after their donation, 2.6% of the 88 participating sperm donors considered an unlimited offspring number acceptable, and 15.9% of the 149 participating oocyte donors considered an unlimited number of donor offspring acceptable (Sydsjö *et al.*, 2014). An American survey conducted in 2014 among members of the Donor Sibling Registry, a registry that facilitates voluntary contact between people genetically related through donor conception, received responses from 176 oocyte donors, 149 sperm donors, 2135 parents raising donor-conceived children, and 419 donor-conceived people. This study found that a majority of respondents from all groups had a preference for having a limit over having no limit in place, with the preference for a limit being strongest among recipients and least pronounced among donors (Nelson *et al.*, 2016). However, this study did not ask for any details on the preferred limit. More recently, a survey among 233 active sperm donors in Denmark and the USA in 2020 found that 18.8% of participants preferred an offspring limit of 10 children or lower, while 48.5% were comfortable with offspring numbers above 100 or considered that the number does not matter. These donors were all counselled and informed that donor anonymity can no longer be guaranteed in light of developments in genetic testing (Pennings *et al.*, 2021). In contrast, a survey among 39 US oocyte donors at the same bank in 2023 found that 41.2% of the participants preferred an offspring limit of 10 children or lower (Lassen *et al.*, 2025).

The first version of this position paper was published for stakeholder review in November 2025. In total, 45 completed review forms were received, including from organizations representing donor-conceived people, families built through donor conception and infertility patients, national fertility societies, researchers and professionals from different disciplines (psychologists, medical specialists, geneticists, ethicists), and gamete banks. A list of the participants in the stakeholder review is provided in Supplementary Data File S2. All comments received are published along with responses in a review report, available on the ESHRE website. The vast majority of the reviews were in favour of establishing international donor offspring limits. With the exception of gamete banks, who have a financial interest in this matter, and a few individual experts, most reviewers supported a limit of 15 families or lower as the ultimate aim.

### Practical aspects on compliance and enforcement

To monitor compliance with an international limit, authorities would have to assess whether donors have donated at different institutions within and across countries and combine information on the number of families resulting from these different donations. Currently, there is no centralized international registry with these data, and building an international donor registry can be expected to be a challenging, costly, and time-consuming task. Even if a mechanism to monitor donations within and across different institutions and countries is established, limits can be undermined by the possibility of private donations outside of any institution. While this practice is illegal in some countries and should be avoided due to the legal and medical risks described earlier in this position paper, it is not possible to fully prevent it. An additional challenge for monitoring donor offspring limits lies in the need to follow up on treatment outcomes, since not all treatments with donor gametes result in the birth of a child. If only live births are counted that are proactively reported back to gamete banks and clinics, this may lead to the limit being exceeded in case of low reporting levels. Therefore, it is important to consider how families are counted and to ensure that establishments follow up with patients and report back on treatment outcomes to gamete banks or donor registries.

### Legal aspects

A worldwide limit could legally only be achieved through an international treaty, which would require immense political efforts and only have limited possibilities of enforcement. Instead of an international treaty, an EU-wide limit could represent a feasible first step towards an international limit. Such a limit could be implemented as an additional standard for offspring protection under the EU SoHO Regulation, which could be added through a delegated act in line with Article 58(16). When determining the feasibility of EU legal action, it is important to carefully consider the principles of conferral, subsidiarity, and proportionality. A discussion on the application of these three principles is provided in Supplementary Data File S3.

## ESHRE position and policy recommendations

ESHRE considers that the wellbeing of donor-conceived people should have the highest priority when determining limits on the number of donor offspring per gamete donor. Such limits will have a direct effect on donor-conceived people by determining the number of genetic relations that they might have. While the same is true for gamete donors, donors consciously choose to engage in gamete donation, whereas donor-conceived people cannot be asked for consent on the conditions of their conception. Although definitive evidence of harm for donor-conceived people and donors is limited, ESHRE considers that the existing evidence and the views expressed by stakeholders sufficiently support a precautionary approach seeking to minimize potential harm for donor-conceived people and donors. Nonetheless, the wellbeing of prospective parents also needs to be taken into account and balanced, including considerations on access to treatment. Based on these considerations, we have formulated the following position and recommendations:

### Setting an international limit

- ESHRE is in favour of setting an international limit on the number of offspring per donor to reduce feelings of uncertainty, overwhelm, and commodification among donor-conceived people and donors and provide clarity and security of expectations to all parties. As there is currently no international body that can impose a worldwide limit, we call on the EU to start by introducing an EU-wide limit.
- Any limit on the number of offspring per donor should be set in terms of the number of families rather than the number of individual children born. (In this position paper, a family is considered as a context where multiple children are raised together as siblings. A definition of the term family that provides legal certainty in all cases needs to be set in legislation.) This is important to ensure that recipients can always use their cryopreserved embryos, independent of how many children were born in other families. Moreover, families may prefer using gametes from the same donor for all their children for various reasons, such as the consideration that this may strengthen the sibling bond or the wish to minimize the number of donors and donor siblings with whom relations may need to be managed if their children are interested in seeking contact. Allowing families to keep using gametes from the same donor gives them reproductive agency over their own family planning.
- Donors should be offered the possibility to set a lower limit for the number of families that can be created with their own gametes than the one that is mandated by regulations and be asked whether they consent to the distribution or export of their gametes to other countries.
- ESHRE is not in favour of restricting the cross-border exchange of gametes. Particularly within the EU, where people, services, and goods can move freely between countries, we call for a harmonized EU-wide framework rather than focusing on national borders.
- ESHRE recommends a phased introduction of an EU-wide family limit, starting with a higher limit to avoid sudden disruptions in access to treatments with donor gametes and give gamete banks time to adjust their operations. This limit should be gradually reduced, alongside the monitoring of the implications for different stakeholders, including the impacts of the limit on access to treatments with donor gametes. As a starting point, ESHRE proposes an EU-wide limit of 50 families per donor. While considered feasible for immediate implementation, a 50-family limit would already constitute a major reduction compared to the current situation, where some gamete banks apply self-imposed limits as high as 75 families or have no self-imposed limits at all (European Sperm Bank). ESHRE ultimately supports a maximum international limit of 15 families or lower per gamete donor. During the transition period, further research should be conducted on the ultimate ideal limit, and if needed, the proposed 15-family limit should be revised in line with updated knowledge.
- The proposed international limits are not intended to replace national limits, but rather to complement them. Current national limits should still be upheld if they are lower. Gamete banks that already operate below a 50-family limit are recognized for their good practice.
- To avoid large age gaps between donor siblings and between donor-conceived people and donors, gametes should no longer be distributed to new families after 20 years following the first donation (as this can be considered the timespan of a generation). Similar to the proposed family limit, the 20-year limit should be reviewed after implementation with a view to potential reduction of the timespan allowed.
- Until obligatory international limits are introduced, ESHRE calls on gamete banks to self-impose the recommended limits and on fertility clinics to only collaborate with banks that apply the proposed limits.
- The introduction of an EU-wide limit and any reductions of the limit should be implemented in such a way that it does not impact ongoing treatments where donor gametes were already assigned to patients or embryos were already created.
- Efforts should be made to increase the donor pool to avoid shortages that reduce affordable access to treatments with donor gametes and increase the need for patients to use unregulated private donations. Similar measures should be taken to those set out in the SoHO Regulation for the supply continuity of critical SoHO, e.g. encouraging public and non-profit involvement in donation programmes and putting in place donor recruitment strategies, including communication campaigns and education programmes.

### Monitoring compliance

- The EU should set a legal basis for an EU-wide donor registry to monitor donations within the EU, including gamete imports from non-EU countries. It should be explored whether this registry could be used for secondary purposes, such as supporting tracing in case of the diagnosis of a serious genetic condition or giving access to some non-identifying information to donors on their offspring and to donor-conceived people on the donor and donor siblings, such as the year of birth, country of birth, and sex assigned at birth. The option of using this registry to provide access for donor-conceived people to find out the identity of the donor (where allowed in the applicable national legislation) could also be explored.
- In case it is not feasible to create an EU-wide donor registry, it should be ensured that each country has a national registry in place that allows monitoring adherence to the limit in the donations of a single donor made in the country, independent of the country of use of the gametes.
- Before an EU-wide donor registry is in place, gamete banks should be obliged to comply with the limit in their own distribution of gametes. This requirement should also apply to banks outside of the EU as a pre-requisite to be able to import gametes into the EU.
- Donors should be required to provide a signed declaration disclosing all previous donations and confirming their commitment to refrain from making private (unregulated) donations. If the number of families to whom gametes from previous donations were distributed is not known, banks/clinics should not accept donors for further donations, as there is a significant risk of exceeding the set limits.
- Where monitoring systems across banks are available, e.g. within a group of banks from the same operator or through an existing national registry, it should be ensured that the limit per donor is upheld across all banks that can be monitored together.
- In the absence of knowledge on whether a live birth took place, the number of families to whom gametes were distributed should be counted as families with a potential live birth to ensure that the family limit is never exceeded. Only if it is known that a donation did not result in a live birth and a family has no more cryopreserved embryos created with gametes from the donor should that family be removed from the count and further gametes be distributed to another family. This system is already in place in many gamete banks, who require clinics to purchase a ‘pregnancy slot’ for each recipient, which is then only released if it is known that the donation did not lead to a child and there are no remaining embryos. Clinics are encouraged to follow up on the pregnancy outcomes of treatments with donor gametes and report back to banks to allow for the release of pregnancy slots where possible.

## Supplementary Material

deag108_Supplementary_Data_File_S1

## Data Availability

No new data were generated or analysed in support of this research.
