# Supplementary material for: ESHRE position paper: international limits on the number of offspring per gamete donor
Source: Hum Reprod. 2026 Jul 8;41(8):1270–6. doi: 10.1093/humrep/deag108 (PMC13429872; doi:10.1093/humrep/deag108)
Supplement: deag108_Supplementary_Data_File_S1 [file deag108_supplementary_data_file_s1.docx]

# Supplementary Data File S1: National donor offspring limits in European countries

| Country | Maximum number of children per sperm donor | Maximum number of children per oocyte donor |
| --- | --- | --- |
| Austria | 3 | 3 |
| Belgium | Children in 6 families | Children in 6 families |
| Bulgaria |  |  |
| Croatia | 3 | 3 |
| Cyprus | 1 | 1 |
| Czech Republic |  |  |
| Denmark | Children in 12 families | Children in 12 families |
| Estonia | Children of 6 different women | Children of 6 different women |
| Finland | Children of 5 different women | Children of 5 different women |
| France | 10 | 10 |
| Germany | Children in 15 families (recommended) | Oocyte donation not allowed |
| Greece | 12 | 12 |
| Hungary | Yes (value not disclosed) |  |
| Iceland | Children in 2 families (in practice, not by law) | Children in 2 families |
| Ireland | Children in 4 families (recommended) | Children in 4 families (recommended) |
| Italy | 10 | 10 |
| Latvia | 3 | 3 |
| Liechtenstein | Not known | Not known |
| Lithuania | 5 | 5 |
| Luxembourg | 3 | Oocyte donation not allowed |
| Malta | 1 | 1 |
| Norway | Children in 6 families |  |
| Poland | 10 | 10 |
| Portugal | Children in 8 families |  |
| Romania | 5 (recommended) | 5 (recommended) |
| Slovakia | 3–5 (recommended) | 3–5 (recommended) |
| Slovenia | Children in 2 families | Children in 2 families |
| Spain | 6* | 6 |
| Sweden | Children in 6 families | Children in 6 families |
| Switzerland | 8 | Oocyte donation not allowed |
| The Netherlands | Children in 12 families |  |
| United Kingdom | Children in 10 families | Children in 10 families |
| * Spain: including children of the family of the donor | | |

Sources: [1] & results of a joint survey of the Council of Europe and ESHRE currently under preparation for publication

Note: National limits can be defined and applied in different ways (e.g., only for treatments in residents of the country, for all treatments in the country including in cross-border patients, or for all treatments including treatments in other countries in case of gamete exports). This information was not collected in the surveys, so it is not included in the table.

# Supplementary Data File S2: List of participants in the stakeholder review

| **Reviewer** | **Country** | **Participation on behalf of (if any)** |
| --- | --- | --- |
|  | UK | Donor Conception Network |
| Anna Coundley | UK | Human Fertilisation and Embryology Authority (HFEA) |
| Aoife Campbell | Ireland |  |
| Kaj Rydman  Kamal Ahuja | UK | London Sperm Bank |
| Mary Wingfield | Ireland |  |
| Menno Hofman | Netherlands |  |
| Hannah Kennedy | UK | Bristol Centre for Reproductive Medicine (BCRM) |
|  |  | European Society of Human Genetics (ESHG) |
| Melinda Ildiko Mitranovici | Romania |  |
|  | UK | Donor Conceived UK |
| Katharina Horn  Jennifer Sutholt | Germany | SolomüJer Deutschland e. V. |
|  | Denmark | Cryos International Sperm & Egg bank |
| Karla Turner | UK |  |
| Nicolás Prados Dodd | Spain | Spanish Society of Reproductive Biology (ASEBIR) |
|  | Denmark | The Danish Business Association for Egg and Sperm  Donation (BFSD) |
| Sara Dalla Costa | Italy |  |
| Evangelini Evgeni | Greece | Cryogonia Cryopreservation Bank |
| Lexi Ellingsworth  Helen Gibson | UK | Stop Surrogacy Now UK  Surrogacy Concern |
| Annemieke Hoek | Netherlands |  |
| Hayley King | UK | Paths to Parenthub |
| Rebecca Kerner | Australia | Australian and New Zealand Infertility Counsellors Association (ANZICA) |
| Lisa Ransome | Australia |  |
|  | US | California Cryobank LLC, and CCB DEB, LLC dba Donor Egg Bank USA, both CooperSurgical, Inc. (CSI) Companies |
| Meurig Gallagher | UK | Association of Reproductive and Clinical Scientists |
| Ties van der Meer | Netherlands | Stichting Donorkind |
| Catherine Metzler-Guillemain | France | Centre d’Etude et de Conservation des Oeufs et du Sperme (CECOS) Federation |
| Juan José Guillén | Spain |  |
| Marieke Verberg | Netherlands | Dutch Society of Obstetrics and Gynecology (NVOG), specifically the Reproductive Medicine pillar |
| Stéphane Viville | France |  |
| Marilyn Crawshaw | UK |  |
| Leah Gilman | UK |  |
| Michael Scholtes |  |  |
| Nwora Melie | UK | Fertility Exeter, Royal Devon University Healthcare Trust |
| Francoise Shenfield |  |  |
| Keerti Singh | Barbados |  |
| Laura Roos  Karin Wadt | Denmark | Department of Clinical genetics, Rigshospitalet, Copenhagen |
| Juan Manuel Moreno-Moya | Norway |  |
| Hana Konečná | Czech Republic |  |
| Valentina Berruti | Italy | La Cicogna Distratta. Istituto di Alta Formazione e di Psicoterapia Familiare di Firenze (Institute of Advanced Training and Family Psychotherapy, Florence) |
| Laura Fernández Olmedilla | Spain |  |
| Astrid Indekeu | Belgium |  |
| Anita Fincham |  | Fertility Europe |
| Linda Savolainen | Sweden | Board of Health and Welfare |

# Supplementary Data File S3: Discussion of EU legal principles

## Conferral:

Based on the principle of conferral, the EU can only act within the limits of the competences that EU Member States have conferred upon it in the treaties [2]. Article 168 of the Treaty on the Functioning of the European Union (TFEU) states that “a high level of human health protection is to be ensured in the definition and implementation of all Union policies and activities”. Moreover, it assigns a legal competence to the EU to adopt measures setting high standards of quality and safety of organs and substances of human origin. The recently adopted EU Regulation 2024/1938 on standards of quality and safety for substances of human origin intended for human application (SoHO Regulation) is based on this article and already includes a provision on the enforcement of national donor offspring limits in article 58(10), demonstrating that the topic can be considered within the scope of EU legal competence. The SoHO Regulation further states that “*[…] SoHO entities shall mitigate any other avoidable risk to the health, including where related to the protection of dignity, in accordance with national law, of SoHO recipients or of offspring from medically assisted reproduction […]* ” (article 58(9)), clearly going beyond the mere protection from physical health risks in its scope.

## Subsidiarity:

The principle of subsidiarity states that the EU shall only intervene when the objectives of an action cannot be sufficiently achieved by the Member States [3]. Currently, donor offspring limits are often set within the national legislations of EU Member States. If all EU Member States adopted a national limit, the sum of these limits would effectively represent an EU-wide donor offspring limit. However, there is an inherent limitation to the maximum limit that can be reached in this manner. Even if each EU Member State implemented a national limit of only one family per donor, this would imply a 27-family limit at EU level, far surpassing the preferred limit of stakeholder groups. Moreover, there are challenges to the enforcement of national limits when donors, recipients, and gametes move between Member States. Recipients frequently travel across borders for treatments with donor gametes, in which case their country of residence has no possibility to enforce the offspring limits in their country. Moreover, when gametes are exchanged between Member States, the competent authority that regulates the distributing gamete bank does not have a mandate to enforce the national regulations of the receiving country. This challenge was demonstrated in a recent case where a Danish sperm bank distributed sperm from the same donor to at least 14 different fertility clinics in Belgium, leading to a number of families created with sperm from this donor that far surpassed the Belgian national limit of six families [4]. In conclusion, national action is unlikely to be sufficient for effectively limiting the number of offspring per donor in the EU.

## Proportionality:

Under the principle of proportionality, EU measures must be suitable and necessary to achieve the desired end and must not impose a burden on the individual that is excessive in relation to the objective sought to be achieved [5]. Setting an EU-wide donor offspring limit is likely suitable to reduce feelings of uncertainty, overwhelm and commodification among donor-conceived people and to increase the clarity and security of expectations of all parties. The need for donor offspring limits is already recognised in the national legislations of most EU Member States (see Appendix 1), but as described above, an EU-wide limit is necessary due to the movement of people and gametes across the EU. A donor offspring limit might impose a burden, particularly on individuals who cannot become pregnant without the use of donor gametes. However, the aim to provide access to treatment for these individuals must be balanced against the welfare of the offspring. Access to treatment can also be enhanced through measures to increase the donor pool rather than through unlimited use of gametes from the same donor, thereby respecting the mental health and dignity of offspring.

A proportionality analysis also needs to consider the impact of an EU-wide donor registry on the privacy of donors, recipients and donor-conceived people. An EU-wide donor registry is suitable to monitor adherence to an EU-wide donor offspring limit, and it is also necessary, since national registries cannot account for the movement of donors, recipients and gametes between Member States. In the EU, there are high data protection standards set in the General Data Protection Regulation (GDPR) that ensure that personalised data is handled responsibly and with as little risk for the data subjects as possible. In accordance with the GDPR, an EU-wide donor registry would need to be set up in line with the principle of data minimisation, collecting only those data that are strictly necessary to monitor adherence to the offspring limit. These data would most likely need to include identifiable data on donors to ensure that a donor cannot surpass the limit by donating at several institutions. However, gamete donation is based on a free and voluntary decision and donors who have strong privacy concerns can always decide against donating. In contrast, a strong desire for a child may cause recipients to pursue treatment with donor gametes despite substantial privacy concerns, and donor-conceived people have no choice at all about the data that is recorded about them at birth. It should be explored whether fully anonymous data on these groups could be sufficient to achieve the objective to monitor offspring numbers.

# References

1. Calhaz-Jorge, C., et al., *Survey on ART and IUI: legislation, regulation, funding, and registries in European countries—an update.* Human Reproduction, 2024. **39**(9): p. 1909-1924.

2. European Union. *Principle of conferral*. Available from: <https://eur-lex.europa.eu/EN/legal-content/glossary/principle-of-conferral.html>.

3. European Union. *Principle of subsidiarity*. Available from: <https://eur-lex.europa.eu/EN/legal-content/glossary/principle-of-subsidiarity.html>.

4. *52 children in Belgium were conceived sperm from a donor with cancer-causing gene*. vrtnws 2025; Available from: <https://www.vrt.be/vrtnws/en/2025/05/30/50-children-in-belgium-were-conceived-sperm-from-a-donor-with-ca/>.

5. European Union. *Principle of proportionality*. Available from: <https://eur-lex.europa.eu/EN/legal-content/glossary/principle-of-proportionality.html>.
